# CAR-T cells in systemic sclerosis

**DOI:** 10.1007/s10067-025-07554-1

**Published:** 2025-07-04

**Authors:** Lazaros I. Sakkas, Chistina Katsiari, Vasiliki Syrmou, Ian C. Chikanza

**Affiliations:** 1 Faculty of Medicine, School of Health Sciences, University of Thessaly, Larissa, Greece; 2 Department of Rheumatology and Clinical Immunology, Faculty of Medicine, School of Health Sciences, University of Thessaly, Larissa, Greece; 3 Pediatrics Department, University of Zimbabwe, Harare, Zimbabwe; 4 International Arthritis and Hypermobility Centre, Harley Street Clinic, London, UK

**Keywords:** Adverse effects, B cells, Bispecific monoclonal antibodies, T cells

## Abstract

Systemic sclerosis (SSc) is a complex disease with extensive fibrosis, microvasculopathy, and autoantibodies. Activation of the adaptive immune system involving T cells, B cells, and macrophages along with microvasculopathy is considered early events in disease pathogenesis. Today, immunosuppressives, including anti-B cell biologics, form the foundation of SSc treatment; however, significant therapeutic needs remain unmet. Autologous chimeric antigen receptor (CAR)-T cells, targeting B cell CD19 and inducing deep depletion of B cells, were tested in a few patients with SSc, leading to improvement of skin score, arthritis, digital ulcers, and inflammatory changes in heart and lungs. However, regression of lung fibrosis remains a major therapeutic challenge. In addition, autologous CAR-T cells are a personalized treatment that requires a specialized setting, is not readily available, is costly, and is associated with serious adverse effects, including cytokine release syndrome, neurotoxicity, and increased risk of infection. New developments in CAR T cell technology, including off-the-shelf allogeneic CAR T cells, bispecific CAR-T cells, and CAR NK cells, are being tested in patients with progressive refractory SSc.

**Table:** 

Key Points • *There is unmet therapeutic need for systemic sclerosis(SSc).* • *Autologous CAR-T cells targeting CD19 showed efficacy in SSc, but are a personalized, expensive and not readily available treatment.* • *Off-the-shelf allogeneic CAR-T cells and CAR-NK cells are being evaluated in SSc.*

Key Points

• *There is unmet therapeutic need for systemic sclerosis(SSc).*

• *Autologous CAR-T cells targeting CD19 showed efficacy in SSc, but are a personalized, expensive and not readily available treatment.*

• *Off-the-shelf allogeneic CAR-T cells and CAR-NK cells are being evaluated in SSc.*

Systemic sclerosis (SSc) is a complex autoimmune disease characterized by excessive and widespread fibrosis, microvasculopathy, and autoantibodies (autoAbs). The disease, apart from skin fibrosis, frequently manifests with digital ulcers and internal organ impairment, which lead to heavy disease burden, poor health-related quality of life, and high mortality [1].

The pathogenesis of the disease is incompletely understood but involves T cells, B cells of the adaptive immune system, and cells of the innate immune system [2, 3]. T cells are of Th2 type producing profibrotic cytokines interleukin (IL)−4 and IL-13. In skin lesions, T cells express early activation antigens and expand in response to antigen(s) [4, 5]. T cells also exhibit cytotoxicity to endothelial cells [6], whereas circulating follicular helper T cells, increased in SSc, enhance B cell antibody production through IL-21 [7]. The activation of the adaptive immune system in SSc is evident by the presence of many autoAbs, some of which are included in the classification criteria for the disease, such as anti-DNA topoisomerase I (ATA, formerly anti-Scl70), anticentromere antibodies (ACA), and anti-RNA polymerase III antibodies (ARPA). B cells are involved in disease pathogenesis through profibrotic autoAbs, pro-inflammatory cytokines (IL-6), and antibody-dependent cellular cytotoxicity [8, 9]. On the other hand, immunosuppressive regulatory B cells are impaired [10]. In recent years, interferon alpha from the innate immune system has emerged as a more prominent factor in disease pathogenesis [11]. Chemokine (C-X-C motif) ligand 4(CXCL4) binding to DNA/RNA induces interferon (IFN)α production in plasmacytoid dendritic cells (pDCs) and activates B cells, thus providing a link between the innate and the adaptive immune system [12–14].

Microvasculopathy and immune system activation, involving T cells and B cells, are generally considered early events leading to the introduction of immunosuppressive therapies which now form the basis of SSc treatment today [15, 16]. Apart from conventional general immunosuppressives affecting T cells and B cells, such as methotrexate, used primarily for skin fibrosis, cyclophosphamide (CyP), and mycophenolate mofetil (MMF), used for SSc-associated interstitial lung disease (SSc-ILD), more targeted immunosuppressives are used for SSc-ILD. These include biologic agents, such as rituximab, a monoclonal antibody (moAb) against CD20, present mostly on B cells, and tocilizumab, a moAb against IL-6 receptor. Newer antifibrotic agents, such as nintedanib, have been added to SSc-ILD treatment. Combinations of biological agents, nintedanib, and conventional immunosuppressives can also be used for SSc-ILD [17, 18]. Autologous hematopoietic stem cell transplantation (AHSCT) is reserved for refractory severe and progressive disease, although a combination of biologics with immunosuppressive/biologics may provide a more favorable outcome compared to AHSCT [17, 18].

Despite the availability of immunosuppressives, Nintedanib, and vasodilators, significant therapeutic gaps remain for patients with SSc, particularly in managing interstitial lung disease (ILD), skin calcinosis, digital ulcers (DUs), and gastrointestinal tract involvement. Chimeric antigen receptor (CAR) T cell therapy offers hope for a more effective treatment for SSc.

## CAR-T cells

In CAR-T cells, the CAR structure consists of an extracellular antigen-binding domain, a hinge region, a transmembrane domain, and an intracellular domain. The extracellular binding domain is composed of variable heavy and light chains of a moAb connected with a flexible linker peptide. The hinge region and the transmembrane domain originate from IgG1, CD28, or CD8, and the intracellular domain contains intracellular activating CD3z, fused with intracellular co-stimulatory CD28 and 4-1BB domains, leading to increased activation and enhanced effectiveness of CAR-T cells [19]. The CAR gene is introduced into T cells usually via viral transfection. Initially, a moAb against CD19 antigen was selected, as CD19 is expressed across a broader range of B cells than the CD20 antigen, spanning from pre-B cells to memory B cells and plasmablasts, except plasma cells.

For autoimmune diseases, a more logical approach would be to target only autoreactive B cells producing autoantibodies, if these autoAbs are proven to be pathogenic. These autoreactive B cells bear B cell antigen receptor (BCR) that recognizes the relevant autoantigen. Chimeric autoantibody receptor (CAAR)-T cells featuring an extracellular domain with an extracellular autoantigen could selectively bind to and eliminate autoreactive B cells. This strategy is particularly logical for diseases driven by pathogenic autoantibodies, such as pemphigus vulgaris, and myasthenia gravis [20]. Of note, BCR is expressed on all B cells from pre-B cells to memory B cells, except plasmablasts and plasma cells.

## Autologous CAR-T cell therapy for systemic sclerosis

Rituximab may not achieve complete B cell depletion, while its efficacy in rheumatoid arthritis was found to depend on the degree of B cell depletion [21]. Based on this and the effectiveness of CAR T cells in B cell malignancies, the rationale was established for the introduction of more potent anti-B cell therapies, specifically CD19-targeted, CAR-T cells (CD19.CAR-T cells), for SSc patients.

Thus far, there is limited experience of autologous CD19.CAR-T cell therapy in SSc with few case reports or small case series [22–24]. In a patient with diffuse cutaneous SSc (dcSSc) of less than 2-year duration, exhibiting skin, lung, and heart fibrosis and anti-RNA polymerase II antibodies (subunit RP 11), CAR-T cells improved many aspects of the disease [22]. At 3 months post-CAR T cell infusion, there was improvement in arthritis, skin score, and heart inflammation on positron emission tomography (PET)/CT scan. Forced vital capacity (FVC) percent predicted remained stable during the 6-month follow-up, whereas diffusion lung capacity of carbon monoxide (DLCO) percent predicted improved. RP11 disappeared during the 6-month follow-up and B cells reappeared after day 80 post-treatment [22]. In another patient with severe SSc, CAR-T cells increased FVC and DLCO, decreased inflammatory lung lesions on PET/CT scan, and decreased serum C-reactive protein (CRP) and ATA, as well as circulating immune complexes [25]. Auth et al. published a series of 6 patients with dcSSc [24]. FVC and DLCO remained stable during a follow-up period of up to 600 days after CAR-T cell infusion. On chest CT, ground-glass opacities decreased but the overall reticular pattern remained stable, whereas on PET/CT, metabolically active changes decreased in the lungs and heart. High pro-B-type natriuretic peptide (pro-BNP) levels, present in one patient, quickly decreased. Antinuclear antibody (ANA) levels rapidly disappeared, and ATA levels exhibited a slower decrease over 200 days, whereas ARPA levels disappeared initially but reappeared after day 400 post-treatment. The intensity of Raynaud’s phenomenon decreased but not its frequency. Digital ulcers were healed, and hand function improved [24]. Natural killer (NK) cells expressing Fcγ-receptor IIIA(FcγRIIIA) were less activated in parallel with the clinical improvement of the patient [26]. Also, Scl70-containing immune complexes that activated FcγRIIIA decreased over time, whereas Scl70-containing immune complexes activated FcγRIIIA-expressing NK cells in a dose-dependent manner [26]. Several trials are under way to assess the efficacy and safety of CAR-T cells in SSc (NCT 06347718, NCT 06328777, NCT 06549296, NCT06548607).

However, in a scleroderma mouse model, CD19.CAR-T cells exacerbated fibrosis. In a Fra-2 transgenic scleroderma mouse model, CD19.CAR-T cells combined with anti-CD20 moAb led to increased lung fibrosis and elevated right ventricular systolic pressure [27]. Nevertheless, these results should be interpreted with caution, as scleroderma mouse models do not accurately reflect the pathogenetic events in human SSc.

## CAR-T cell challenges

Treatment with autologous CD19.CAR-T cells presents several challenges. Firstly, it is a multistep process that involves leukapheresis to obtain T cells from a patient, ex-vivo activation and viral transfection of T cells to introduce the CAR gene, and finally CAR-T cell expansion for about 2 weeks. There are also quality assays to be performed for each batch of CAR-T cells. Furthermore, before the CAR-T cell infusion, patients receive lymphodepleting chemotherapy with fludarabine and/or cyclophosphamide [28]. Secondly, adverse effects may be serious. Cytokine release syndrome (CRS) and immune effector cell–associated neurotoxic syndrome (ICANS) are apparently due to widespread and strong activation of CAR-T cells and occur frequently in B cell malignancies. CRS is graded from grade 1 (mild fever and fatigue) to grade 4 (life-threatening organ impairment) [29]. In B cell malignancies, severe CRS (grade ≥ 3) was found in 10–25% and ICANS in 22–38% of patients [30], but in autoimmune diseases, CRS and ICANS are generally mild [31]. In a series of 15 patients with autoimmune rheumatic diseases (8 with systemic lupus erythematosus [SLE], 4 with SSc, and 3 patients with idiopathic inflammatory myositis [IIM]), CRS (grade 1) occurred in 11 of 15 patients (none with SSc) and mild ICANS in one patient with IIM [23]. In another study with 6 patients with SSc, CRS was absent or mild (3 patients with grade 1 and one patient with grade 2) (24). The risk of lymphodepletion with myelotoxicity and B cell depletion is also of concern. B cell reconstitution in autoimmune diseases occurs around 4 months after CAR T cell infusion. B cell aplasia develops after one infusion of CD19.CAR-T cells that may last several years in B cell malignancies but fortunately around 4 months in autoimmune diseases [32, 33]. The viral vectors with CAR transgenes used for T cell transduction permanently intergrade CAR DNA into patient’s genome [34]. This caries a risk of insertional mutagenesis resulting in transformation of T cells and long-term cancer development, as has been reported in autologous CAR-T cell therapy for cancer [35].

The EBMT recently has published a position statement and clinical recommendations for cellular therapies, including CAR-T cells, in autoimmune diseases [36].

Treatment with autologous CAR-T cells is expensive and not readily available. In addition, CAR-T cells may differ from patient to patient. Efforts have been made to develop off-the-shelf CAR-T cells from unrelated donors. These allogeneic CAR-T cells could provide immediate availability and consistent quality. However, there are challenges involved, including graft-versus-host disease (GVHD) and allogeneic cell rejection by host T cells and NK cells. Editing genes involved in GVHD and graft rejection, such as knocking out HLA-class I (HLA-I) and native T cell receptor (TCR) genes with clustered regularly interspaced short palindromic repeats (CRISPR) and CRISPR-associated protein 9 (CRISPR/Cas9), minimizes this risk. By reducing HLA-I expression on CAR-T cells, allogeneic rejection by host T cells is reduced but allogeneic rejection by host NK cells increases: When HLA-I antigens are not present on allogeneic cells, host NK cells do not receive inhibitory signals by immunoglobulin-like receptors (KIRs). As a result, NK cells receive activating signals which prompt them to target and kill the allogeneic cells [37]. Allogeneic CD19.CAR-T cells were administered in two patients with SSc and markedly improved lung inflammation and fibrosis on high-resolution CT (HRCT) scan and myocardial inflammation and fibrosis on cardiac magnetic resonance imaging (MRI). In addition, they greatly improved skin tightness associated with disappearance of B cells and decrease of transforming growth factor (TGF)β levels in skin biopsies [38]. The percentage of allogeneic CD19.CAR-T cells decreased from 31.5% at 30 days post-treatment to 4.4% at 6 months, whereas ATAs nearly disappeared [38].

The authors, apart from deleting HLA-class I, HLA-class II, and TCRα, selected CD3-negative cells to avoid GVHD by allogeneic T cells [38].

Allogeneic NK cells, genetically modified to express CD19-targeting CAR (CD19.CAR-NK cells), were used for B cell malignancies [39–41]. Allogeneic cord blood unit (CBU)-derived NK cells were transduced with a retroviral vector expressing genes that encoded CD19-targeting CAR, IL-15 (to improve their proliferation and function), and inducible caspase-9(iC9)-based suicide gene (as a safety switch to pharmacologically activate iC9 suicide gene and eliminate these cells in case of toxicity) [39, 40]. CAR-NK cell infusion resulted in efficacy comparable to CAR-T cells in B cell malignancies and absence or low levels of CRS, ICANS, and GVHD [40, 41]. In one third of patients, IgG decreased to less than 400 mg/dL within 90 days [40]. CAR-NK cells persisted at low levels for at least 12 months [39]. When using banked CBU for CAR-NK cell production, the best predictors for superior outcome in B cell malignancies were the number of nucleated red blood cells in CBU (≤ 8 × 10^7^) and the time from collection to cryopreservation (≤ 24 h) [40]. Allogeneic CD19.CAR-NK cells, derived from a healthy donor, are being evaluated in immune-mediated diseases including SSc (NCT06733935).

## Future perspective

The field of CAR T cells is rapidly evolving. CAR-encoding mRNA can be used to transfect T cells ex vivo via electroporation or in vivo via nanoparticles injection to transiently express CAR [28]. Repeated infusion of autologous CD8 + T cells transfected *ex-vivo* with mRNA to express B cell maturation antigen (BCMA)-targeting CAR in patients with myasthenia gravis improved outcome measures and was well-tolerated [42]. mRNA-based CAR technology avoids the risk of insertional mutagenesis and can be used without lymphodepleting chemotherapy. Therefore, this strategy is particularly appealing for children with autoimmune diseases.

Induced pluripotent stem cells may solve the problem of allogeneic cell source, as they may provide a continuous supply of cells for off-the-shelf CAR-T cells and/or CAR-NK cells to ensure consisted quality [43] (NCT05859997). Induced pluripotent stem cell–derived CD19.CAR-NK cells are being evaluated in patients with refractory B cell–mediated autoimmune diseases including dcSSc (NCT06255028). Thus far, CAR T cells targeting CD19 + B cells improve the inflammatory component of ILD, but their effects on fibrosis in SSc-ILD are not clearly evident. New generation CAR T cells, dual-target CAR T cells targeting CD19 and BCMA, have already been used in a phase I trial in SLE [44, 45] and are being tried in clinical trials in SSc (NCT 06775912, NCT06794008, NCT06503224). Dual CAR-T cell specificity against B cells and T cells (CD19.CD3E CAR-T cells) is also being studied in SSc (NCT06373081). Gene editing by CRISPR/Cas9, inhibiting specific molecules such as TCR and HLA-I and HLA-II, was used to diminish GVHD and enhance the persistence of allogeneic CAR-T cells. Long-term toxicity, including secondary malignancies, is of concern [46], and various modifications are being developed, including safety switches and mRNA-based CAR technology [47]. The allogeneic CAR-T cells are expected to be rejected due to residual allogeneity and thus avoid the risk of long-term transformation and cancer development [35].

CAR-NK cells from allogeneic banked CBUs are an alternative approach to CAR T cells for autoimmune diseases, including SSc. There is no need for leukapheresis, and hundreds of CAR-NK cell doses can be manufactured from a single banked CBU for future use.

CD19.CAR-T cells experience thus far reinforces the idea that effective B cell depletion appears to be at the center of promising future treatments. Bispecific moAbs may offer similar efficacy to CAR T cells in autoimmune rheumatic diseases [48, 49]. Obinutuzumab, an anti-CD20 monoclonal antibody which kills more B cells than rituximab, was tried in a patient with limited cutaneous SSc (lcSSc) and chronic lymphocytic leukemia and resulted in elimination of cutaneous calcinosis and improvement of FVC [50, 51]. Blinatumomab, a bispecific anti-CD3/CD19 moAb, which binds CD3 of TCR and engages cytotoxic T cells to lyse B cells has been tried in a patient with severe SSc and was very effective in reducing B cells, skin tightness, and Raynaud’s phenomenon [49, 52].

It should be emphasized that any therapy targeting immune cells should be applied early in the disease process, when the pathogenic role of these cells is more pronounced [2, 3, 53].

In conclusion, the therapeutic armamentarium for SSc is likely to expand and include cellular therapies, and the prognosis of SSc is likely to be more optimistic.
